# Socioeconomic inequalities in the place of death in urban small areas of three Mediterranean cities

**DOI:** 10.1186/s12939-020-01324-y

**Published:** 2020-12-03

**Authors:** Andreu Nolasco, Manuel Fernández-Alcántara, Pamela Pereyra-Zamora, María José Cabañero-Martínez, José M. Copete, Adriana Oliva-Arocas, Julio Cabrero-García

**Affiliations:** 1 Research Unit for the Analysis of Mortality and Health Statistics, Department of Community Nursing, Preventive Medicine, Public Health and History of Science, University of Alicante, Alicante, Spain; 2 Department of Health Psychology, University of Alicante, Alicante, Spain; 3 Department of Nursing, University of Alicante, Alicante, Spain; 4 Institute for Health and Biomedical Research of Alicante (ISABIAL- FISABIO Foundation), Alicante, Spain

**Keywords:** Mortality, Inequalities, Small-area analysis, Palliative care, Residential facilities, Place of death

## Abstract

**Background:**

Dying at home is the most frequent preference of patients with advanced chronic conditions, their caregivers, and the general population. However, most deaths continue to occur in hospitals. The objective of this study was to analyse the socioeconomic inequalities in the place of death in urban areas of Mediterranean cities during the period 2010–2015, and to assess if such inequalities are related to palliative or non-palliative conditions.

**Methods:**

This is a cross-sectional study of the population aged 15 years or over. The response variable was the place of death (home, hospital, residential care). The explanatory variables were: sex, age, marital status, country of birth, basic cause of death coded according to the International Classification of Diseases, 10th revision, and the deprivation level for each census tract based on a deprivation index calculated using 5 socioeconomic indicators. Multinomial logistic regression models were adjusted in order to analyse the association between the place of death and the explanatory variables.

**Results:**

We analysed a total of 60,748 deaths, 58.5% occurred in hospitals, 32.4% at home, and 9.1% in residential care. Death in hospital was 80% more frequent than at home while death in a nursing home was more than 70% lower than at home. All the variables considered were significantly associated with the place of death, except country of birth, which was not significantly associated with death in residential care. In hospital, the deprivation level of the census tract presented a significant association (*p* < 0.05) so that the probability of death in hospital vs. home increased as the deprivation level increased. The deprivation level was also significantly associated with death in residential care, but there was no clear trend, showing a more complex association pattern. No significant interaction for deprivation level with cause of death (palliative, not palliative) was detected.

**Conclusions:**

The probability of dying in hospital, as compared to dying at home, increases as the socioeconomic deprivation of the urban area of residence rises, both for palliative and non-palliative causes. Further qualitative research is required to explore the needs and preferences of low-income families who have a terminally-ill family member and, in particular, their attitudes towards home-based and hospital-based death.

## Background

Interest in the study of decision-making regarding the place of death has increased in recent decades [1, 2]. Dying at home is the most frequent preference of patients with advanced chronic diseases [3–6], their caregivers [4], and the general population [4], and so it has become an indicator of the quality of palliative care [7]. However, up until today, most deaths continue to occur in hospitals [6, 8].

Place of death predictors are traditionally grouped into three categories: disease-related factors, individual factors, and environmental factors [2]. Research on these predictors through death certificates has usually examined a country’s global information, or that of a region, a municipality, or a specific population subgroup in relation to individual factors such as age [6, 7, 9, 10], sex [6, 7, 9, 10], educational level [7, 11], marital status [6, 10], place of residence in the rural or urban context [6, 7] or the patient’s own preferences [2]. Similarly, the relationship with disease factors, like the diagnosis of the patient’s pathology [6–8, 12–14] and environmental factors such as the availability of family support and home care resources [15–17], as well as other more generally related to or derived from local laws or health policies, has also been examined [6, 18].

In addition, recent studies have also explored the relationship between place of death and aggregate indicators of socioeconomic deprivation [11, 19] with the areas of greatest deprivation being those with the highest hospital mortality rates. Furthermore, various studies seem to indicate that social differences have a lesser effect on the place of death when measured at the individual level (for example, educational level) than when measured at the aggregate level (commonly by means of deprivation at the area level) [19]. In this regard, an adequate instrument to measure inequalities is a deprivation index (DI), like those used in the MEDEA and INEQ-CITIES studies on social inequalities and risk of death [20–22], that combine a set of indicators such as the percentages of unemployed, temporary workers, low educational level of different population groups, or manual workers and allow classifying small geographic areas according to their level of socioeconomic deprivation [23].

In Spain, although some retrospective studies have included indicators of deprivation with respect to the place of death, they have only done so for some specific pathologies [24–26]. No studies of the relationship between these indices of deprivation and place of death for general mortality and according to different causes of death have been carried out as far as this team is aware. Knowledge of these aspects, especially in urban settings, where the majority of the population in Europe and Spain concentrates, would allow the adoption of organizational and guidance measures for health services, particularly those related to palliative care and end-of-life care.

Therefore, the objective of this study was to analyse the socioeconomic inequalities in the place of death among the deaths in the large cities of the Valencian Community (Alicante, Castellón, and Valencia) during the period 2010–2015, using levels of deprivation by small areas of the cities, and also to assess if such inequalities are different depending on palliative (oncological, non-oncological) or non-palliative causes of death.

## Methods

### Study design

This is a cross-sectional study of the resident population in the cities of Alicante, Castellón, and Valencia aged 15 years or over whose death took place between 2010 and 2015. These cities are located on the Mediterranean coast, in the Valencian Community, with an average annual population during the study period of 333,198 inhabitants in Alicante, 177,784 in Castellón, and 794,874 in Valencia.

### Data sources and variables

All deaths of residents in those cities during the study period were included in the analysis. Data obtained from the Valencian Community Mortality Registry, anonymised and included in the Medical Death Certificate - Statistical Death Bulletin (Spanish initials: CMD-BED), were used. For each city, deaths were geo-referenced and assigned to their resident census tract (CT) using the address included in the CMD-BED.

The response variable was the place of death. The Spanish registry of deaths includes five possible categories for this variable: home, hospital, residential care, place of work, or other, and the place may be left blank (not recorded). Deaths in the first three places were the focus of this analysis.

The explanatory variables included in the CMD-BED, were: sex (male, female); age (15–64, 65–74, 75–84, 85 and over); marital status (not declared, single, widowed, separated, married); place of birth (Spain, another country) and basic cause of death coded according to the International Classification of Diseases, 10th revision (ICD-10). As this research is based on retrospective anonymized administrative data, the approval of the ethical committee is not necessary for its implementation in Spain.

### Causes of death

Deaths were classified, according to the cause of death, as:

1. Deaths due to ‘conditions needing palliative care’ (CNPC), according to the classification suggested by Murtagh et al. [27], slightly modified by Gomes et al. [10], which includes the following ICD-10 codes: malignant neoplasm, C00-C97; heart disease: I00-I52 (excluding I12 and I13.1); cerebrovascular disease, I60-I69; renal disease, N17, N18, N28, I12, I13,1; liver disease, K70-K77; respiratory disease: J06-J18, J20-J22; J40-J47, J96; neurodegenerative disease: G10, G20, G35, G12,2, G90,3, G23,1; Alzheimer’s disease, dementia, and senility: F01, F03, G30, R54, and HIV/AIDS: B20-B24. These causes of death susceptible to palliative care were further subdivided into ‘oncological conditions needing palliative care’ (OCNPC, including only malignant neoplasm) and ‘non-oncological conditions needing palliative care’ (NOCNPC, other deaths from CNPC).
2. Deaths from any other condition not needing palliative care (CnotPC).

Thus, the result is the variable ‘type of cause’ with 3 categories: OCNPC, NOCNPC, CnotPC.

### Socioeconomic level

In each city, a deprivation index (DI) was calculated for each CT from the following indicators: unemployment, manual workers, temporary employees, insufficient instruction in young people (16 to 29 years old), and insufficient instruction in general, all of them in percentage and obtained from the Spanish Population and Housing Census of 2011. These indicators have been used in the coordinated national project MEDEA to construct a deprivation index through a principal components analysis based on census data in the main Spanish cities [23]. The index used in this study was developed within the framework of the MEDEA III project (third edition of the coordinated MEDEA project).

The DI values of each CT, in each city, were classified by percentiles: 10 (P_10_), 25 (P_25_), 75 (P_75_), and 90 (P_90_), according to the methodology described in Oliva-Arocas et al. (2020) [28] that classifies the CT into five levels of deprivation (DL) according to its value: (DL1, DI values below P_10_; DL2, DI values between P_10_ and P_25_; DL3 DI values between P_25_ and P_75_; DL4 DI values between P_75_ and P_90_ and DL5, values DI higher than P_90_). This classification was defined, according to the objective of the study, to identify and quantify the most extreme inequality, that between the most socioeconomically favoured areas (DL1) and the most deprived areas (DL5).

### Statistical analysis

Frequencies and percentages were calculated for the following variables: death place (home, hospital, and residential care), cause of death (CNPC, OCNPC, NOCNPC, CnotPC), and deprivation level (DL). To compare the frequencies of death between places of death, the excesses of probability of death (‘odds’) in hospital and residential care were calculated dividing the percentage of death in each location by the percentage of death at home. The Nelson approximation was used in order to calculate the 95% confidence intervals (95% CI) [29].

Multinomial logistic regression models were adjusted in order to analyse the association between the place of death and the DL, with the place of death (home, hospital, or residential care) as the response variable and the DL as the explanatory. Simple and adjusted models taking into account the rest of the sociodemographic variables were estimated. In addition, the existence of different socioeconomic inequalities according to the type of cause was surveyed by including in the model an interaction term between the DL and the type of cause. Finally, in all the models, the reference category was domicile (D), estimating the ‘odds ratio’(OR), and its corresponding 95% CI, as a measure of association. The SPSS statistical program version 25 was used, with a 0.05 level to establish statistical significance.

## Results

From 2010 to 2015, there were 67,521 deaths among the resident population in the cities under study, of which 67,200 (99.5% of the total) occurred in the population aged 15 or over. Of these, 876 (1.3%) could not be assigned to their census tract of residence due to the unavailability of a valid residence address or that it did not belong to the city. Of the 66,324 deaths, 60,748 occurred at home, hospital, or residential care facilities, and therefore they were used in the data analysis. Of these, 49,021 were related to CNPC.

### Differences between death in hospital and residential care versus home

A total of 58.5% of deaths occurred in hospitals, 32.4% at home, and 9.1% in residential care (see Table 1).

**Table 1:** Percentages, frequencies (*n*) and Odds of death in each location compared to death at home, according to the categories of the variables studied. Alicante, Castellón and Valencia. 2010–2015

VARIABLE	Percentages of death according to place of death^**a**^			***n***	Odds of death in each location compared to death at home^**b**^					
	Home	Hospital	Resid. care		Hospital			Residential care		
					Odds	95%IC		Odds	95%IC	
**TOTAL**	32.4	58.5	9.1	60,748	1.806	1.774	1.837	0.281	0.273	0.289
**CITY**										
Alicante	31.5	60.7	7.9	14,316	1.927	1.859	1.998	0.251	0.235	0.268
Castellón	32.3	57.6	10.1	7,286	1.783	1.696	1.876	0.313	0.288	0.340
Valencia	32.8	57.9	9.4	39,146	1.765	1.727	1.804	0.287	0.276	0.297
**SEX**										
Men	30.5	63.8	5.7	29,112	2.092	2.040	2.145	0.187	0.177	0.197
Women	34.2	53.6	12.2	31,636	1.567	1.530	1.606	0.357	0.344	0.370
**AGE**										
15–44	16.5	81.2	2.3	919	4.921	4.134	5.859	0.139	0.088	0.220
45–64	23.4	75.0	1.6	6,876	3.205	3.031	3.390	0.068	0.056	0.083
65–84	29.7	63.9	6.3	27,498	2.152	2.096	2.209	0.212	0.201	0.223
≥ 85	38.3	47.3	14.4	25,455	1.235	1.202	1.268	0.376	0.362	0.391
**MARITAL STATUS**										
Not included	26.3	62.4	11.3	1,997	2.373	2.143	2.627	0.430	0.368	0.502
Single	27.1	59.2	13.7	5,694	2.185	2.057	2.320	0.506	0.464	0.551
Widowed	35.0	51.5	13.5	25,652	1.471	1.433	1.511	0.386	0.371	0.401
Divorced	21.3	72.3	6.4	2,347	3.394	3.072	3.750	0.300	0.250	0.361
Married	32.5	63.9	3.6	25,058	1.966	1.914	2.019	0.111	0.103	0.119
**COUNTRY OF BIRTH**										
Spain	32.5	58.3	9.2	59,176	1.794	1.762	1.826	0.283	0.275	0.292
Other	27.4	66.2	6.4	1,572	2.416	2.159	2.703	0.234	0.188	0.290
**DEPRIVATION LEVEL**^**c**^										
DL1	39.8	52.7	7.5	4,907	1.324	1.249	1.404	0.188	0.169	0.211
DL2	34.0	54.2	11.8	9,957	1.594	1.527	1.664	0.347	0.325	0.371
DL3	31.7	59.0	9.2	30,997	1.861	1.816	1.907	0.290	0.278	0.303
DL4	31.0	61.4	7.6	8,971	1.981	1.892	2.073	0.245	0.225	0.267
DL5	29.4	63.2	7.3	5,916	2.150	2.031	2.275	0.248	0.223	0.276
**CAUSE OF DEATH**										
No palliative	22.0	69.8	8.1	11,727	3.173	3.035	3.316	0.368	0.342	0.397
Palliative oncologic	31.6	65.5	2.9	18,044	2.073	2.008	2.139	0.092	0.084	0.100
Palliative no oncologic	36.8	50.1	13.1	30,977	1.361	1.329	1.395	0.356	0.343	0.369

^a^Percentages were calculated over the total frequency with declared place of death

^b^Odds (location/home) = Percentage of death in this location/Percentage of death at home

^c^DL: Deprivation level of the census track of residence based on the deprivation index (DI). DL1: DI < P_10_; DL2: P_10_ ≤ DI < P_25_; DL3: P_25_ ≤ DI < P_75_; DL4: P_75_ ≤ DI < P_90_; DL5: DI ≥ P_90_; P_q_ = Percentile q

The percentage of death in hospitals ranged from a minimum of 47.3% in people aged 85 and over to a maximum of 81.2% in people aged 15 to 44 years. At home, it went from 16.5% in people aged 15–44 to 39.8% in people residing in the DL1 areas (the areas with better socioeconomic status). For residential care, the death rates oscillated between 2.3% in subjects aged 15 to 44 years and 14.4% in those over 85 years of age. Death in hospital was 80% more frequent than at home (Odds for hospital vs. home = O_H/D_ = 1.806), while death in the nursing home was more than 70% lower than at home (Odds for residential care vs. home = O_R/D_ = 0.281).

When comparing hospital with domicile (home), the O_H/D_ were significantly greater than 1 (*p* < 0.05) for any category of the explanatory variables, with the highest excesses of death in men, residents of the city of Alicante, aged between 15 and 44 years old, divorced, and born in another country. Death in hospital was significantly more frequent for CnotPC, followed by OCNPC and finally NOCNPC. Regarding the socioeconomic level of the area of residence, a clear trend was observed in excesses of death in hospital vs. home, from the better off level, DL1 (O_H/D_ = 1.324) to the worst off one, DL5 (O_H/D_ = 2.150).

For residential care, the O_R/D_ was always significantly (*p* < 0.05) less than 1, with lower mortality in men, residents in Alicante, aged between 45 and 64 years, married, and born in another country. Compared to domicile, death in residential care was lower for OCNPC, with NOCNPC and CnotPC presenting similar deficits. The socioeconomic level of the area of residence did not present a clear trend, with low values both in favoured levels such as DL1 and high deprivation levels such as DL5.

**Fig. 1:**
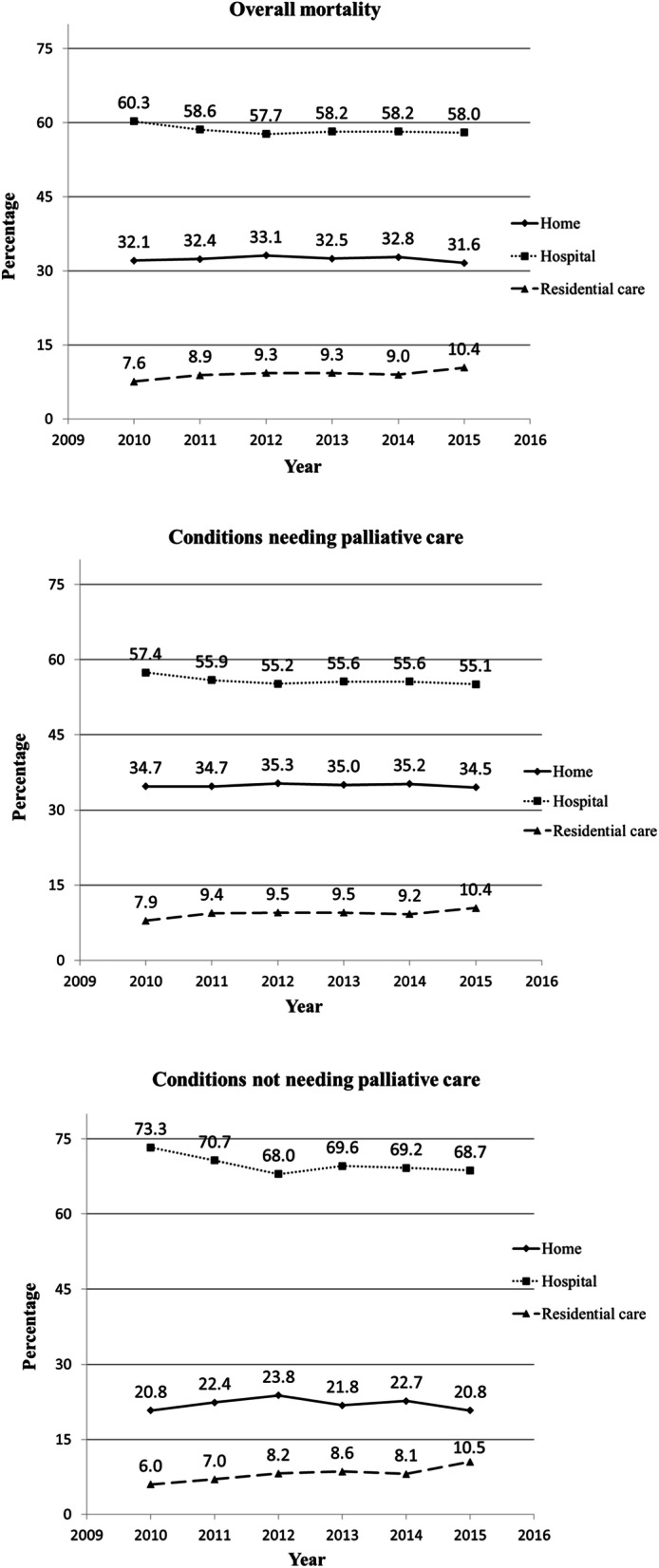
Percentage of death by place and year. Alicante, Castellón and Valencia. 2010–15

Regarding overall mortality, Fig. 1 shows both a slight decrease of deaths in hospital and a slight increase of deaths in residential care homes from the beginning to the end of the period with statistically significant differences (*p* < 0.001) for these two settings. As well, there is a slight increase in residential care deaths for CnotPC. Deaths at home remained stable over time.

### Specific conditions needing palliative care

Table 2 shows the percentages and Odds of death by location for deaths from specific CNPC and the rest of the causes.

**Table 2:** Percentages, frequencies and odds of death in each location compared to death at home according specific palliative/non-palliative causes. Alicante, Castellón and Valencia. 2010–2015

CAUSE OF DEATH	Percentages of death according to place of death^**a**^			Frequency of death	Odds of death in each location compared to death at home^**b**^					
	Home	Hospital	Resid. care		Hospital			Residential care		
					Odds	95%IC		Odds	95%IC	
**TOTAL**	32.4	58.5	9.1	60,748	1.806	1.774	1.837	0.281	0.273	0.289
Malignant neoplasm	31.6	65.5	2.9	18,044	2.073	2.008	2.139	0.092	0.084	0.100
Heart disease	44.0	43.9	12.1	13,232	0.998	0.962	1.035	0.275	0.260	0.291
Cerebrovascular disease	28.1	62.8	9.0	4145	2.235	2.086	2.395	0.320	0.285	0.360
Renal disease	33.4	55.2	11.4	1,202	1.653	1.460	1.871	0.341	0.281	0.414
Liver disease	21.4	75.0	3.6	917	3.505	2.990	4.107	0.168	0.116	0.243
Respiratory disease	25.8	65.7	8.5	4,602	2.547	2.381	2.723	0.329	0.294	0.369
Neurodegenerative disease	41.9	45.1	12.9	904	1.076	0.936	1.238	0.308	0.250	0.379
Alzheimer’s. dementia and senility	39.0	37.1	23.9	5,755	0.951	0.897	1.009	0.613	0.573	0.655
HIV/AIDS	8.6	85.5	5.9	220	9.942	6.202	15.936	0.686	0.339	1.389
Other non-palliative causes	22.0	69.8	8.1	11,727	3.173	3.035	3.316	0.368	0.342	0.397

^a^Percentages were calculated over the total frequency with declared place of death

^b^Odds (location/home) = Percentage of death in this location/Percentage of death at home

Comparing death in hospital vs. home, heart disease was the only cause with O_H/D_ < 1, although not significant. The rest presented O_H/D_ significantly higher than 1 (*p* < 0.05). Among the CNPC, death from HIV/AIDS had the highest odds of death in hospital vs. home, followed by liver disease. Death from malignant neoplasm was located in an intermediate O_H/D_. The rest of the CnotPC had a large excess of death in hospital.

Regarding death in residential care, all causes presented O_R/D_ significantly lower than 1 (*p <* 0.05). The low O_R/D_ value for malignant neoplasm stood out, followed by liver disease. The CnotPC reached an intermediate mortality deficit in residential care.

### Association between the place of death and level of deprivation

Table 3 shows the crude and adjusted OR that estimate the association between the place of death and the explanatory variables. All the variables considered were significantly associated with the place of death, in the simple and multivariate analysis models, except country of birth, which was not significantly associated with death in residential care.

**Table 3:** Association^a^ between place of death and studied variables. Odds Ratios (OR) for each place of death vs. reference category (home). Alicante, Castellón and Valencia. 2010–2015

VARIABLE	Hospital						Residential care					
	Crude OR	95%CI^**b**^		Adjusted OR	95%CI^**b**^		Crude OR	95%CI^**b**^		Adjusted OR	95%CI^**b**^	
**CITY**												
Alicante	1.09	1.05	1.14	1.13	1.08	1.18	0.87	0.81	0.94	0.89	0.83	0.97
Castellón	1.01	0.95	1.07	1.05	0.99	1.11	1.09	0.99	1.19	1.12	1.02	1.23
Valencia	1.00			1.00			1.00			1.00		
**SEX**												
Men	1.33	1.29	1.38	1.20	1.15	1.25	0.53	0.49	0.56	0.89	0.83	0.96
Women	1.00			1.00			1.00			1.00		
**AGE**												
15–44	3.97	3.33	4.74	3.49	2.91	4.19	0.37	0.23	0.58	0.43	0.27	0.69
45–64	2.59	2.44	2.76	2.41	2.25	2.59	0.18	0.15	0.22	0.33	0.27	0.41
65–84	1.74	1.68	1.81	1.69	1.62	1.76	0.57	0.53	0.61	0.88	0.82	0.94
≥ 85	1.00			1.00			1.00			1.00		
**MARITAL STATUS**												
Not included	1.21	1.09	1.34	1.36	1.22	1.51	3.86	3.25	4.58	3.37	2.83	4.01
Single	1.11	1.04	1.19	1.14	1.06	1.22	4.56	4.08	5.09	4.03	3.59	4.53
Widowed	0.75	0.72	0.78	1.06	1.01	1.11	3.47	3.21	3.76	2.49	2.27	2.72
Divorced	1.72	1.56	1.91	1.43	1.28	1.59	2.70	2.22	3.28	3.62	2.96	4.43
Married	1.00			1.00			1.00			1.00		
**COUNTRY OF BIRTH**												
Other country	1.35	1.20	1.51	1.16	1.03	1.31	0.82	0.66	1.03	0.94	0.75	1.18
Spain	1.00			1.00			1.00			1.00		
**DEPRIVATION LEVEL**^**c**^												
DL5	1.50	1.38	1.62	1.39	1.28	1.51	1.07	0.93	1.23	1.16	1.01	1.34
DL4	1.42	1.32	1.53	1.36	1.27	1.47	1.05	0.92	1.19	1.10	0.97	1.25
DL3	1.32	1.24	1.41	1.27	1.19	1.35	1.09	0.98	1.21	1.17	1.05	1.30
DL2	1.12	1.05	1.21	1.09	1.01	1.17	1.32	1.18	1.49	1.35	1.20	1.52
DL1	1.00			1.00			1.00			1.00		
**CAUSE OF DEATH**												
Non-palliative	2.33	2.22	2.45	2.30	2.18	2.42	1.04	0.96	1.13	1.03	0.95	1.12
Palliative oncologic	1.53	1.47	1.59	1.17	1.12	1.22	0.26	0.24	0.29	0.35	0.32	0.39
Palliative non-oncologic	1.00			1.00			1.00			1.00		

^a^Estimation using multinomial regression model with main effects simple and adjusting for all the variables

^b^95CI: 95% confidence interval for the OR

^c^DL: Deprivation level of the census track of residence based on the deprivation index (DI). DL1: DI < P_10_; DL2: P_10_ ≤ DI < P_25_; DL3: P_25_ ≤ DI < P_75_; DL4: P_75_ ≤ DI < P_90_; DL5: DI ≥ P_90_; P_q_ = Percentile q

The multivariate model with all variables had indices pseudo-R2 Cox and Snell = 0.1034 and Nagelkerke = 0.1240. The adjusted ORs showed that the level of deprivation of the CT presented a significant association (*p* < 0.05) and increased from the lowest DL to the highest one. That is, the probability of death in hospital vs. home increased as the deprivation level raised. In addition, the highest probability of death in hospital vs. home was associated with residing in the city of Alicante, male sex, younger age, marital status different from married, and place of birth outside of Spain. The probability of death in hospital vs. home was higher for CnotPC, followed by OCNPC, vis-à-vis the NOCNPC.

The level of CT deprivation was also significantly associated with death in residential care, but with lower ORs than in the case of death in hospital and with a diffuse pattern, since both highly deprived and less deprived CTs presented excesses of the probability of death over the reference level (DL1). Regarding the other variables, the highest probability of death in residential care vs. home occurred in Castellón, in relation to old women, of any marital status other than married, and without significant association with the country of birth. Only OCNPC presented a significant deficit in deaths.

The estimated ORs of association between DL and place of death represent a measure of the level of inequality in the probability of death according to the level of deprivation of the area of ​​residence. To check if these inequalities could be different depending on the cause of death (OCNPC, NOCNPC, CnotPC), an interaction term between DL and cause of death was added to the previous multivariate model. The resulting model presented indices pseudo-R2 Cox and Snell = 0.1037 and Nagelkerke = 0.1244, with little variation with the interaction-less model. Furthermore, the interaction term was not significant (*p* = 0.221), meaning that the estimated inequalities are not different according to these cause groupings.

The effect of sex on the relationship between DL and place of death was also checked by adding an interaction term between DL and sex to the main effects model. Nevertheless, the interaction term was not significant (*p* = 0.322) and presented indices pseudo-R2 Cox and Snell = 0.1036 and Nagelkerke = 0.1242, with little variation with the interaction-less model. This suggests that sex does not substantially alter the deprivation effect.

To delve into the causes that presented the highest association between death in hospital and the level of deprivation, multinomial logistic regression models were adjusted for each of the CNPC and also the CnotPC. Table 4 presents the ORs between death in hospital vs. home and level of deprivation, adjusted by the rest of the explanatory variables (city, sex, age, marital status, and country of birth). Mortality due to respiratory disease, Alzheimer’s disease, dementia and senility, and mortality from CnotPC were noticeable for their significant and high association with the DL as compared with mortality by the rest of the causes of death. This effect has contributed to a greater extent to the global association. Mortality from malignant neoplasm and heart disease also presented a significant but lower association with DL than that of the group of all deaths. Regarding the rest of the causes, no significant association with DL was detected.

**Table 4:** Association^a^ between place of death and deprivation level according cause of death. Adjusted Odds Ratios (OR) for death in Hospital vs. reference category (home). Alicante, Castellón and Valencia. 2010–2015

**VARIABLE**	**Death in Hospital vs. Home**														
	**Malignant neoplasm**			**Heart disease**			**Cerebrovascular disease**			**Renal disease**			**Liver disease**		
	**OR**	**95%CI**^**b**^		**OR**	**95%CI**^**b**^		**OR**	**95%CI**^**b**^		**OR**	**95%CI**^**b**^		**OR**	**95%CI**^**b**^	
**DEPRIVATION LEVEL**^**c**^															
DL5	1.34	1.15	1.55	1.27	1.07	1.50	1.11	0.80	1.55	1.19	0.62	2.27	1.38	0.58	3.28
DL4	1.26	1.10	1.44	1.20	1.03	1.40	1.46	1.09	1.96	0.88	0.51	1.50	0.97	0.45	2.08
DL3	1.29	1.15	1.45	1.07	0.94	1.21	1.24	0.97	1.58	0.92	0.58	1.49	0.93	0.46	1.89
DL2	1.06	0.93	1.20	1.00	0.86	1.16	1.01	0.76	1.33	0.95	0.56	1.61	1.15	0.51	2.59
DL1	1.00			1.00			1.00			1.00			1.00		
	**Respiratory disease**			**Neurodegenerative disease**			**Alzheimer's. dementia and senility**			**HIV/AIDS**			**Other non-palliative causes**		
	**OR**	**95%CI**^**b**^		**OR**	**95%CI**^**b**^		**OR**	**95%CI**^**b**^		**OR**	**95%CI**^**b**^		**OR**	**95%CI**^**b**^	
**DEPRIVATION LEVEL**^**c**^															
DL5	2.01	1.46	2.75	1.11	0.57	2.15	1.64	1.24	2.16	1.93	0.28	13.50	1.55	1.26	1.90
DL4	1.52	1.16	2.00	1.66	0.92	2.97	1.70	1.33	2.18	2.30	0.31	17.34	1.65	1.37	1.99
DL3	1.54	1.22	1.95	1.28	0.81	2.04	1.41	1.14	1.74	4.97	0.69	35.92	1.46	1.25	1.71
DL2	1.46	1.11	1.92	0.91	0.54	1.55	1.15	0.90	1.46	3.38	0.33	35.04	0.20	1.00	1.43
DL1	1.00			1.00			1.00			1.00			1.00		

^a^Estimation using multinomial regression model with main effects, adjusting for the rest of the explanatory variables (city, sex, age, marital status and country of birth)

^b^95CI: 95% confidence interval for the OR

^c^DL: Deprivation level of the census track of residence based on the deprivation index (DI). DL1: DI < P_10_; DL2: P_10_ ≤ DI < P_25_; DL3: P_25_ ≤ DI < P_75_; DL4: P_75_ ≤ DI < P_90_; DL5: DI ≥ P_90_; P_q_ = Percentile q

## Discussion

The objective of this study was to analyse the socioeconomic inequalities in the place of death in the large cities of the Valencian Community (Alicante, Castellón, and Valencia) during the period 2010–2015, using levels of socioeconomic deprivation by small areas of the cities. A part of our aims was to assess if such inequalities were different depending on whether the deaths were due to conditions needing palliative care (oncological, non-oncological) or conditions not needing palliative care. The results have highlighted the existence of socioeconomic inequalities in the sense that greater deprivation would clearly increase the probability of death in hospital vs. home. This effect is not so evident in the case of death in a nursing home. There was no evidence to affirm that the estimated inequalities are different according to the different cause of death groupings. As in previous research [13], other sociodemographic variables such as country of birth, sex, age, marital status, or cause of death have also been associated with place of death.

Importantly, the Survey of Care for Patients with Terminal Illness shows that in 2009 the Spanish population (18+ years) preferred to be cared for at home (45.0%), followed by care in a specialized center (31.9%) and only 17.8% would choose a hospital in the case of irreversible disease in terminal phase [30]. This shows the high percentage of the population that prefers to end their days of life at home.

Regarding the excesses of deaths in hospital vs. home, significantly higher values ​​were observed in the most deprived CT, especially in respiratory disease, Alzheimer’s disease, malignant neoplasm, heart disease, and other non-palliative causes. In all cases, an association was observed between living in areas with greater deprivation and probability of dying in hospital, as indicated by previous studies [11, 19, 31, 32]. This is an important result since death at home is usually considered an indicator of the quality of palliative care services [3, 10]. The higher number of deaths in hospital may be related to the difficulty that people living in CT with higher deprivation have to access health resources, which might not reach these patients adequately [11]. Likewise, it is possible that, in line with what other authors suggest, people who live in a place with greater deprivation prefer to die in hospital as compared to home or residential care home [33, 34], as well as other contextual factors that might be at work, such as the difficult economic or labour situation associated with the places of greatest deprivation [19].

Another possible explanation for these results has to do with the care burden and social support to the caregivers. A lower socioeconomic level is associated with a greater burden of care, and a greater difficulty in receiving formal support. The burden of care has been associated, in many cases, with the need for help in daily life tasks, rather than with the specific symptoms presented by the patient [35]. This burden of care can make caregivers prefer to have their family members dying in hospital, where there are more resources and will receive more support to cope with the end of life.

In this regard, a recent qualitative study explored the relationships between social disparities and the burden of care in cancer patients and showed how the social determinants of health such as low income, low education, precarious housing conditions, rurality (associated with difficulty in the access to palliative care) or lack of social support could exacerbate the caregiver’s overload [36]. Likewise, social support is an important variable that can mediate and positively regulate the perceived care burden [37, 38]. Neergaard et al. identified in their review a series of variables related to social support such as living with other family members, having family support, being married, availability of space at home, the region of residence, as well as the caregiver’s sociodemographic variables (age, sex, and relationship with the patient) [19].

Regarding the diagnoses most associated with the different levels of deprivation, a very heterogeneous profile was found. This includes the three trajectories associated with the end-of-life process: advanced cancer (malignant neoplasm), advanced organ disease (heart and respiratory disease), and advanced dementia (Alzheimer’s dementia and senility) [39]. In addition to these CNPC, diagnoses for CnotPC were also significant, and deprivation seems to have an important role. This great variability in the diagnoses found is consistent with studies that indicate that the burden of care is similar among those diagnosed with an oncological process or in cases of diseases not related to cancer [35, 40].

Results regarding the risk of death in residential care home vs. home showed a more complex association pattern, with both high and low deprivation CTs showing an excess probability of death in residential care vs. home. An increase in the number of deaths in residential care facilities can be observed, related to the ongoing aging of the population, as well as an increase in pathologies such as dementia, which can mean that, regardless of the level of deprivation, many people end up dying in residential care homes. Also, the situation of nursing homes in Spain includes both public and private institutions, and so, regardless of the level of deprivation of a person’s CT, it is possible to move to them. In this regard, various studies in countries such as the United States have associated low socioeconomic status with access to poorer quality nursing homes [41, 42].

It is important to highlight that the progressive breakdown of the Spanish health system, in particular primary care and public health, due to the long period of austerity and privatizations (particularly in some regions) [43, 44], plus the overload of care resulting from budget cuts has had a serious impact on the quality of primary care [45]. The consequences of this deterioration have differentially affected the most deprived populations. This may explain why the hospital is the main place of death for many people living in more deprived places.

This work presents a series of limitations among which are those related to the use of data from the CMD-BED, since there may have been undetected errors in the diagnosis or during encoding and transcription. The CMD-BED was not modified during the study period. On the other hand, the CMD-BED in Spain includes a limited number of variables, so it was not possible to consider some of them individually, i.e. the employment situation or the type of work. Instead, the deprivation level variable included information on such variables, considered at the contextual level of the area of ​​residence, and thus, the excess probability of death in one or other locations according to the level of deprivation could reflect both the effect of the individual socioeconomic level as much as the contextual effect of the area of residence.

Another limitation comes from not having geo-referenced all deaths. Nevertheless, only a very small percentage (1.3%), lower than usual in this type of study, was not included. These losses should have had little effect on the results obtained.

It should be borne in mind that the classification in DL would not be the only possible one either. Nevertheless, it responds to the objective of preferentially evaluating the inequality existing between the population groups of greater and lesser deprivation, with consistent results across the different categories used.

Finally, this work did not include preferences about the place of death. Further research is needed to investigate the effect that deprivation may have on the congruence or incongruence between the place where a person wants to die and where death finally occurs.

## Conclusions

The results of this study indicate that the probability of dying in hospital as compared to dying at home, increases as the socioeconomic deprivation of the urban area of ​​residence rises, and this generally happens either for any type of palliative death (oncological and non-oncological) or non-palliative. However, when comparing death in residential care vs. home it can be seen that the effect of the level of socioeconomic deprivation is very limited since only the areas of least socio-economic deprivation (the first level) are slightly associated with a lower probability of death in residential care. While socioeconomic differences in access to formal and informal care may explain the greater probability of death in hospital for people living in areas of greater deprivation, the way these factors influence death in residential care vs. home is largely unknown. Further qualitative research is required to explore the needs and preferences of low-income families who have a terminally-ill family member and, in particular, their attitudes towards home-based and hospital-based death.

## Data Availability

Data is available at the Valencian Community Mortality Registry.

## Abbreviations

CMD-BED: Medical Death Certificate - Statistical Death Bulletin
ICD-10: International Classification of Diseases 10th revision
CT: Census tract
CNPC: Conditions needing palliative care
OCNPC: Oncological conditions needing palliative care
NOCNPC: Non-oncological conditions needing palliative care
CnotPC: Condition not needing palliative care
DI: Deprivation index
DL: Levels of deprivation
DL1: The most socioeconomically favoured areas
DL5: The most socioeconomically deprived areas
D: Domicile
95% CI: 95% Confidence intervals
OR: Odds Ratio
